# Synchronous/Metachronous Prostate Cancer with Other Cancer Sites—The Experience of a Single Center in Romania

**DOI:** 10.3390/medicina61091666

**Published:** 2025-09-14

**Authors:** Gabriela Rahnea-Nita, Mihaela Dumitru, Alexandru Nechifor, Roxana-Andreea Rahnea-Nita, Adrian-Cornel Maier, Liliana Florina Andronache, Andreea Cernea, Alexandru Rebegea, Laura-Florentina Rebegea

**Affiliations:** 1 Specific Disciplines Department, The Faculty of Nursing and Midwifery, “Carol Davila” University of Medicine and Pharmacy, 020021 Bucharest, Romania; gabriela.rahnea-nita@umfcd.ro; 2 M Hospital, 010903 Bucharest, Romania; 3 The Department of Radiotherapy, “Sfantul Apostol Andrei” Hospital, 800578 Galati, Romania; mihaeladumitru11@yahoo.com (M.D.); cernea_georgia@yahoo.com (A.C.); laura.rebegea@ugal.ro (L.-F.R.); 4 The Clinical Department, The Faculty of Medicine and Pharmacy, “Dunarea de Jos” University of Galati, 800008 Galati, Romania; alexandru.nechifor@ugal.ro (A.N.); alexandru.rebegea20@gmail.com (A.R.); 5 The Clinical Department, The Faculty of Medicine, “Carol Davila” University of Medicine and Pharmacy, 020021 Bucharest, Romania; 6 The Preclinical Department, The Faculty of Medicine, “Carol Davila” University of Medicine and Pharmacy, 020021 Bucharest, Romania; liliana.andronache@umfcd.ro

**Keywords:** multiple primary malignancy, synchronous/metachronous prostate cancer, staging, therapeutic management

## Abstract

*Background and Objectives*: The management of patients with multiple primary malignancies (MPMs) is performed by a multidisciplinary team whose purpose is to individualize the treatment plan and to determine the strategies that will improve the patient’s care. The most commonly encountered malignancies are prostate cancer, colorectal cancer, lung, and bladder cancer. Prostate cancer may be synchronous or metachronous with other cancer sites. The risk factors for MPMs are viral infections, genetic factors, environmental factors, and oncological treatments (chemotherapy and radiotherapy). *Materials and Methods*: In this retrospective cohort study, we analyzed 26 patients with either synchronous or metachronous prostate cancer with another cancer site, registered at “Sf. Apostol Andrei” County Emergency Hospital in Galati, Romania, over a 15-year time interval (2010–2025).This paper presents the clinical and therapeutic characteristics and the staging of synchronous/metachronous cancer sites. The stage of the disease was analyzed along with the time interval between the first cancer site and the second or the third cancer site occurrence; the undergone treatment was a multimodal one. *Results*: This paper discusses the analyzed cases and presents the articles published in the specialty literature regarding the stage of synchronous or metachronous prostate cancer with one or more cancer sites. The most common sites are presented, along with the importance of follow-up and a complex therapeutic management depending on the stage of primary tumors. *Conclusions*: This series represents a cohort study regarding synchronous/metachronous prostate cancer with other cancer sites (26 cases over a 15-year time period). This case series adds more experience to the already existing experience in the literature, providing guidelines to clinicians in order to make the right decisions regarding the treatment of this disease.

## 1. Introduction

In order to diagnose multiple primary malignancies (MPMs), it is necessary that the following criteria be met: each tumor must be malignant, it must be different histopathologically, and the possibility that one tumor is the metastasis of another must be ruled out [1].

Multiple primary malignancies (MPMs) can be synchronous or metachronous. Synchronous tumors are those that occur at the same time or within a two-month interval, according to the definition provided by the Surveillance Epidemiology and End Results (SEER) project, or within a 6-month interval, according to the definition provided by the International Association of Cancer Registries and International Agency for Research on Cancer (IACR/IARC) [1,2,3,4].

The incidence of multiple primary malignancies was 1.63% in a study conducted in Taiwan on 109,054 cancer patients, monitored for a period of 25 years. In most patients (70.87%), the second cancer occurred after two months [5].

The risk factors for MPMs are viral infections, genetic factors (BRCA1, BRCA2, HOX B13 mutations, mismatch repair genes, genetic instability, and Lynch Syndrome), chronic inflammation, environmental factors, betel quid chewing habit, chemotherapy, and any history of previous radiotherapy [6,7,8,9,10,11,12,13,14,15].

The general mechanisms involved in the occurrence of synchronous and metachronous prostate cancers are BRCA1 and BRCA2 genetic mutations, TP 53 mutation, genomic instability and MMR gene mutations in Lynch syndrome, any history of previous radiotherapy (radiation-induced cancers—rectal, bladder, or colon cancer occurring after radiotherapy for prostate cancer or the occurrence of prostate cancers after radiotherapy for bladder or rectal cancers), and hormonal therapy with antiandrogens that can alter hormonal balance and induce other types of cancers, such as breast cancer in men, which is very rare, but possible.

The management of patients with multiple primary malignancies (MPMs) is often difficult. It is performed by a multidisciplinary team whose purpose is to individualize the treatment plan and to determine the strategies that will improve the patient’s care [16,17,18,19,20,21].

Prostate cancer occurs most commonly in men aged 45–60 years; it is the second most common type of cancer in men worldwide, has the most common cause related to cancer in Western countries, and ranks fifth worldwide in terms of cancer-related mortality in men [22,23,24,25].

Post-therapeutic evolution is directly influenced by tumor grading and the stage of cancer at the time of treatment initiation.

Prostate cancer has a second metachronous site, i.e. colorectal cancer, thus ranking second in terms of metachronous associations. Head and neck cancer have esophageal cancer as a second metachronous site, thus ranking first in terms of metachronous associations [5,26].

## 2. Materials and Methods

This cohort study was conducted at “Sf. Apostol Andrei” County Emergency Hospital in Galati, Romania. In this retrospective study, we analyzed 26 patients with prostate cancer either synchronous or metachronous with another cancer site, registered at “Sf. Apostol Andrei” County Emergency Hospital in Galati, Romania, over a 15-year time period (2010–2025).

### Inclusion and Exclusion Criteria

**The inclusion criteria were**

- Age > 18 years;
- Patients with a histopathologically confirmed diagnosis of prostate cancer;
- Patients with a histopathologically confirmed diagnosis of a second primary cancer;
- Comprehensive pretherapeutic assessment;
- Patients who signed the informed consent form.

**The exclusion criteria were**

- Patients without a histopathologically confirmed diagnosis of prostate cancer and a second or third primary cancer;
- <6 months of available followup data;
- Patients who did not sign the informed consent form.

**Data collection:**

The patients’ files were reviewed for information regarding the diagnosis, the stage of the disease, the date of the positive histopathological result, and the complex treatment and follow-up of prostate cancer and cancer/cancers with another site. The date of diagnosis was defined as the date of the positive histopathological result. The data were recorded until the date of the last visit or until the time of death.

## 3. Results

The median age of 26 patients was 72.5 years (ranging from 56 to 83 years).

The first tumor site was prostate for 14 patients (53.86%); 3 patients (11.54%) had renal cancer as the first tumor site. The second tumor site was prostate for 10 patients (38.46%), each lung for 4 patients (15.38%), and colon-rectum for 5 patients. The third tumor site was prostate for 2 patients (7.69%), colon-rectum for 2 patients (7.69%) and pancreas for 1 patient (3.85%) (Table 1). The average follow-up period was 84 months (ranging from 12 to 180 months). The follow-up period was calculated from the moment of the diagnosis of the second neoplasm until the end of the study.

The first tumor site occurred between 2010 and 2024. The histopathological diagnosis for the first tumor site was adenocarcinoma in 16 cases (61.54%). For the first tumor, the treatment performed was surgery in 12 cases (46.15%), external beam radiotherapy in 15 cases (57.69%), chemotherapy in 4 patients (15.38%), and hormonal therapy in 10 cases (38.46%) (Table 2).

The second tumor site occurred between 2014 and 2024. The histopathological diagnosis for the second tumor site was adenocarcinoma in 18 cases (69.23%) and papillary carcinoma in 3 cases (2 patients were diagnosed with thyroid cancer and 1 patient was diagnosed with bladder neoplasm).

For the second tumor, the treatment performed was surgery in 16 cases (61.53%), external beam radiotherapy in 10 cases (38,46%), chemotherapy in 6 patients (23.07%), and hormonal therapy in 9 cases (30.77%) (Table 3).

The third tumor sites occurred between 2020 and 2025. The histopathological diagnosis for the third tumor site was adenocarcinoma for all patients. For the third tumor, the treatment performed was surgery in two cases (40%), external beam radiotherapy in three cases (60%), and hormonal therapy in two cases (40%) (Table 4).

The most frequent combinations are shown in Table 5.

The mean time interval between the first and the second tumor site occurrence was 54 months (ranging from 1 to 180 months); 2 cases had synchronous cancers and 24 patients had metachronous neoplasm (Table 6). The mean time interval between the first and the third tumor site occurrence was 98 months (ranging from 24 to 180 months); all five patients had metachronous neoplasm (Table 7). The mean time interval between the second and the third tumor site occurrence was 48 months (ranging from 12 to 84 months), all of them having metachronous cancers (Table 8).

Regarding Gleason score, we have made a comparison between two groups: the first group included patients with first primary prostate cancer and the second group patients with prostate cancer as the second or third neoplasm. This analysis concluded that 3 patients from the first group and 4 patients from the second group had a Gleason score of 6; 6 patients from the first group and 3 patients from the second group has a Gleason score of 7; and 1 patient from the first group and 4 patients from the second group had a Gleason score of 9. A Gleason score of 8 was encountered in three patients in the first group and two patients in the second group (with prostate cancer as the second or third neoplasm); this comparison was not statically significant, *p* = 0.85.

**Figure 1:**
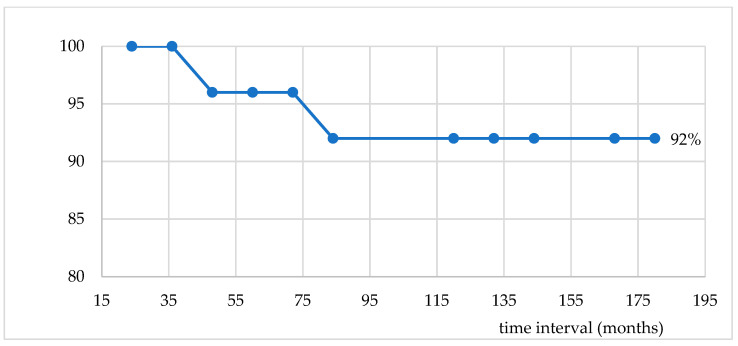
Overall survival.

**Figure 2:**
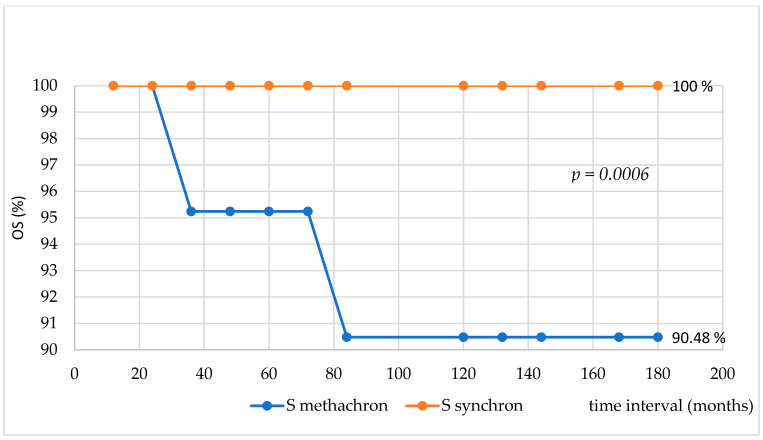
Comparative analysis of survival for patients with metachronous vs. synchronous prostate cancer.

**Survival analysis.** The overall survival (OS) was analyzed with the help of a Kaplan–Meier curve (Figure 1). The OS was 92% at 180 months after a median follow-up period of 84 months (ranging from 12 to 180 months). In our cohort, there were only two individuals with metachronous cancer who died. One died after 36 months from the first diagnosis of neoplasm, and the other one died after 84 months from the same diagnosis. So, all patients with synchronous prostate cancer were alive at the end of the follow-up period; the comparative Fisher test revealed a statistically significant difference between patient survival with metachronous prostate cancer (90.48%) and patient survival with synchronous prostate cancer, *p* = 0.0006 (Figure 2).

**Figure 3:**
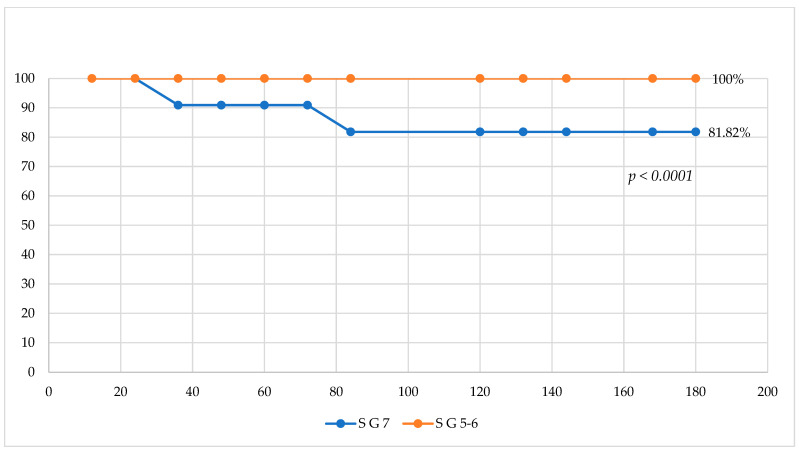
Comparative analysis of survival for patients with Gleason score 7 vs. Gleason scores 5 and 6.

**Figure 4:**
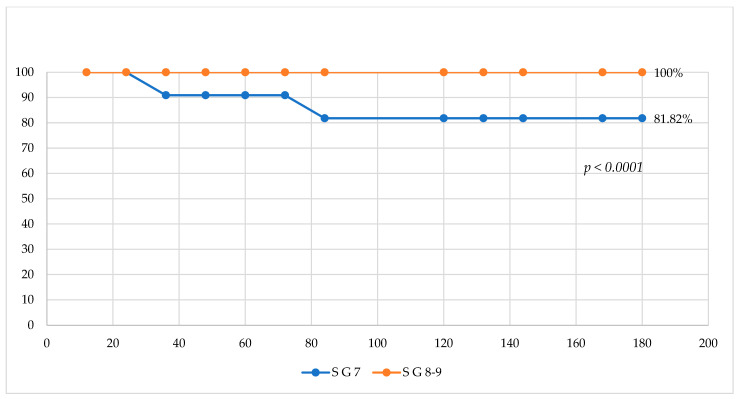
Comparative analysis of survival for patients with Gleason score 7 vs. Gleason scores 8 and 9.

We performed an analysis on the survival rate, using Gleason score (Figure 3 and Figure 4). We grouped all 26 patients into three subgroups: patients with Gleason scores of 5 and 6 in one subgroup (7 cases), patients with a Gleason score of 7 in another subgroup (11 cases), and patients with Gleason scores of 8 and 9 in the third subgroup (8 cases). Analyzing overall survival in a comparative mode, we found that patient survival with a Gleason score of 7 was 81.82% vs. 100% for patients with Gleason scores of 5 and 6, *p* < 0.0001; also, the same situation was found when comparing the OS of patients with a Gleason score of 7 with the OS of patients with Gleason scores of 8 and 9: 81.82% vs. 100%, *p* < 0.0001. The explanation might be the small number of deceased patients, both of whom had a Gleason score of 7.

In order to explore more factors influencing survival, we performed a multiple linear regression test, analyzing the influence of Gleason score and sequencing of prostate tumor (the first, second, or third neoplasia) on overall survival. These analyses revealed that none of these factors influenced survival in any significant way (Table 9).

## 4. Discussion

The survival of cancer patients has improved globally in recent years, thanks to early diagnostic methods, screening, and the development of systemic therapies, targeted therapies, and radiotherapy techniques.

Patients who have survived one neoplastic site occurrence may develop a second or even a third neoplastic site during their lifetime, and the incidence of multiple primary synchronous or metachronous cancers has increased in recent years.

Our cohort of patients is heterogeneous, with various diagnoses and different types of sequencing. Prostate tumors, as a first, second, or third tumor site, were encountered in 50%, 42.31%, and 7.69% of patients, respectively.

In our patients, there were more cases of second and third primary metachronous neoplasms of prostate cancer with a 9 Gleason score than cases of first primary neoplasms of prostate cancer; in these cases, we might interpret this as an increase in the aggressiveness of prostate cancer when it appears as a metachronous second site.

In 8 out of the 26 patients in our study group, the metachronous prostate tumor was associated with another site occurrence, namely, cancer of the urogenital system, as follows: 4 cases of bladder urothelial tumors and 4 cases of Gravitz-type renal tumors.

In our study, the treatment of prostate cancer was determined according to the recommendations of the urologic oncology guidelines: total androgenic blockade or radiotherapy/radical prostatectomy in cases where the tumor is localized.

In 2024, Zhang Y et al. published the case of a patient with synchronous prostate and kidney cancer and revealed that the treatment was surgical (prostatectomy and nephrectomy) for localized tumors, while in synchronous metastasized cancers, the starting point of metastases must be investigated in order to determine an adequate treatment [27].. The patient underwent left nephrectomy, left adrenalectomy, and lymph node dissection of the left renal hilum and retroperitoneum area. The adrenal metastases, the renal hilar lymph nodes, and the retroperitoneal lymph nodes originated in prostate cancer and responded to hormonal treatment. The conclusion was that the staging of the two cancers was essential for the correct therapeutic management [27].

In our study, there were three patients with prostate and renal cancer. In two of them (patient no. 12 and patient no. 26), renal cancer was the primary tumor site, while in one patient (patient no. 25), prostate cancer was the primary tumor site. All three patients underwent surgery for renal cancer. Regarding prostate cancer, patient no. 12 received ADT, while patients nos. 25 and 26 received EBRT and ADT.

In 2023, Bunnag N et al. published the case of an 84-year-old patient with synchronous prostate and lung cancer, revealing that the therapeutic strategy was different from prostate cancer with single-lung metastasis, a situation that is extremely rare in the initial staging of prostate cancer [28].

In 2021, Sarkar S et al. published the case of a patient with synchronous prostate and lung cancer that started with symptoms specific to both lung cancer (cough and hemoptysis) and prostate cancer (difficulty in eliminating urine), within a one-month interval [29].

Four patients in our study had prostate cancer and lung cancer. In all four patients, lung cancer was the second neoplastic site. The patients were tested for genetic mutations and the three of them who were EGFR-, ALK-, and PDL1-negative, underwent systemic treatment. One patient had an EGFR mutation (patient no. 5) and thus underwent a third-generation anti-TKI treatment with Osimertinib, at present exhibiting a stable pulmonary condition. Prostate cancer was treated with surgery in patient no. 2, with EBRT in patient no. 5, with EBRT plus ADT in patient no. 4, and with EBRT plus ADT plus ARPI in patient no. 1.

In 2020, Luqman W et al. published the case of a patient with synchronous prostate cancer and sarcoma with bone metastases, for whom a diagnosis of leiomyosarcoma was made through the biopsy of bone metastasis from the iliac bone [30]. The PET-CT scan may be useful in assessing synchronous cancers with osteolytic metastases in the context of prostate cancer, but, in this patient’s case, osteolytic metastases originating in leiomyosarcoma seemed non-avid to F18-choline but highly avid to F18-FD [30].

There was a patient in our study (patient no. 19) with three cancer sites, two of which were prostate cancer and sarcoma (leiomyosarcoma), the third site being colon cancer. The treatment for prostate cancer was EBRT plus ADT, while the treatment for sarcoma consisted of surgery plus adjuvant EBRT; the colon cancer was treated with surgery.

In 2025, Edfelt E et al. published the largest cohort study on synchronous prostate and rectal cancer [31]. The authors analyzed patients with prostate cancer from the Swedish Cancer Register between 1993 and 2019 and revealed that the incidence of synchronous prostate and rectal cancers has increased over the past 20 years in Sweden (out of 238,252 prostate cancer cases, 594 were synchronous with rectal cancer) [31].

B.U. Sidiqi et al. also conducted a retrospective study over a 5-year period (2017–2022) and identified 10 patients out of 2204 prostate cancer patients with synchronous rectal cancer [32]. The authors suggest a standardized management with upfront chemotherapy, followed by RT inclusive of prostate and rectum, followed by boost via brachytherapy or SBRT in non-candidate patients. Two of the patients did not want to continue with the treatment and both showed progression of the disease. Six out of the ten patients underwent rectal surgery after chemoradiotherapy, and for the two patients who had complete clinical response revealed on MRI, the “Watch and Wait” approach was chosen [32].

In 2023, Sossa J et al. published the case of a patient with metachronous prostate cancer, with the second site being breast cancer, and revealed that the treatment must combine the known therapy for each one of them [33].

In our study, we had a patient with breast cancer as the first tumor site, with prostate cancer as the second site (patient no. 20). The treatment for breast cancer consisted of surgery, radiotherapy, and chemotherapy plus hormone therapy (5 years), while the treatment for prostate cancer consisted of definitive EBRT plus ADT.

Regarding the association between prostate cancer and breast cancer, which occurred in patient no. 20, a study published by Abhyankar N et al., in 2017, concluded that the incidence of prostate cancer in men with a history of breast cancer is similar to the general population [34].

There is a question related to patient no. 20 (a male patient) with a first neoplasia of breast cancer and the second of prostate cancer; the latter occurred within a 3-year interval. Might this case have a hormonal component?

Family history is also an important risk factor in prostate cancer. There is evidence that prostate and breast cancers can occur together in some patients, the hereditary breast cancer-related gene 2 (BRCA2) being identified to contribute to these conditions [35,36,37,38]

BRCA2 mutations significantly increase the risk of both prostate and breast cancer in men. While men generally have a low risk of developing breast cancer, BRCA2 mutations raise that risk substantially, and they also increase the likelihood of developing prostate cancer, potentially at a younger age and with more aggressive forms.

The association of breast cancer and prostate cancer (patient no. 20) is very rare, and in terms of sequencing, most case reports show the occurrence of prostate cancer as the primary neoplasm, and breast cancer occurs more frequently as the second cancer site in a metachronous association. There have been some published cases about synchronous occurrence [39]. We do not have BRCA testing available in this case.

In 2022, Millican J et al. published the experience of a single facility in Western Sydney, Australia, regarding 15 patients with synchronous/metachronous prostate and rectal cancer, over the period between 1 January, 2008, and 1 January, 2020, revealing that there was an increase in the incidence of the two cancer types over time, these two ranking second and third after lung cancer [40]. The factors involved in the increase in the incidence were obesity, reduced fiber intake, increased consumption of alcohol and red/processed meat, and a sedentary lifestyle. The results of patients who underwent prior radiotherapy for prostate cancer are revealed. Patients with prior prostatectomy also benefited from neoadjuvant chemotherapy and treatment. Moreover, the management of synchronous prostate and rectal cancer is presented [40].

In our study, there were two patients with rectal and prostate cancer (patient no. 17 and patient no. 22), with rectal cancer being the second cancer site. The treatment for rectal cancer consisted of surgery in one patient, whereas in the second patient, surgery and EBRT plus chemotherapy were performed. Patient no. 17 underwent EBRT plus ADT (36 months) for prostate cancer, while patient no. 22 underwent EBRT upon relapse plus ADT. Patient no. 5, as previously mentioned, had three cancer sites, namely, prostate, lung, and rectum.

In 2024, Hoshi S et al. published three cases of prostate cancer with metachronous urothelial cancer (both ureteral and renal pelvis), revealing that in the follow-up process of patients with urological cancer, it is important to monitor not only the progression of the first urological cancer but also the possibility of occurrence of a second urological cancer [41]. Moreover, the authors mention the fact that while bladder cancer has a higher incidence than the metachronous prostate cancer, in terms of urothelial cancer, the ratio is limited [41].

We had four patients with bladder and prostate cancer in our study; in two of them (patient no. 14 and patient no. 21), bladder cancer was the first site, while in the other two patients (patient no. 3 and patient no. 7), prostate cancer was the first site. The treatment for bladder cancer consisted of TUR-V plus CHT instillation in one patient, TUR-V plus EBRT upon relapse in one patient, surgery in one patient, and TUR-V plus CHT in one patient. The treatment for prostate cancer consisted of EBRT plus ADT + ARPI in one patient, EBRT plus ADT in one patient, and surgery in two patients.

In 2021, Zhao T et al. published the case of a patient with triple metachronous cancer sites of the kidney, prostate, and squamous cell carcinoma of the lung, and it revealed that genetic analysis is essential in the diagnosis of triple cancers [42]. The identification of aberrant mutations that affect the PI3K-Akt pathway across different tumors and the treatment with a combination of PI3K inhibitor BKM120 and AKT inhibitor MK2206 prolonged the patient’s survival rate by more than one year [42].

In our study, we had five patients with three metachronous cancer sites, with prostate cancer being the third tumor site in two patients (patient no. 18 and patient no. 25). In the other three patients, the third cancer site was the rectum, colon, and pancreas.

Regarding the association between thyroid cancer, renal cancer, and prostate cancer found in one of the patients in our study, namely, patient no. 18, a study in the specialized literature published by Bellini et al. in 2022, reveals that there is a common association between thyroid cancer and other cancers such as prostate cancer, breast cancer, colorectal cancer, and renal cancer [35]. No correlation has been identified between the development of thyroid and kidney cancer and the performance of radiotherapy or chemotherapy [35].

There is a question arising from this analysis, namely, whether there is a causality connection between radiotherapy and the second cancer appearance (radio-induced cancer) in those cases in which the second cancer occurred after the performance of radiotherapy for the first cancer site; this might be a possible cause for the following patients: no. 17 (prostate cancer in 2014 and rectum cancer in 2024) and no. 21 (bladder cancer in 2015 and prostate cancer in 2024).

Case 17 developed upper rectal cancer 10 years after radiotherapy for prostate cancer. Surgery followed by chemotherapy was performed for rectal cancer. It is possible that this cancer was radiation-induced.

Prostate cancer survivors treated with prior radiotherapy are at increased risk of developing rectal cancer compared to those treated with prostatectomy [43].

There are studies that do not show a statistically significant association between rectal cancer and a history of radiotherapy to the prostate [44].

Other authors have found a risk of developing bladder cancer after radiotherapy for prostate cancer [44].

Regarding patient no. 21, he developed prostate cancer 9 years after undergoing radiotherapy for bladder cancer. Also, in this case, there is a possibility that it was a radiation-induced cancer.

There is very little information in the literature on the occurrence of prostate cancer after radiotherapy for bladder cancer; most studies have investigated the incidence of bladder, rectal, and colon cancers after radiotherapy for prostate cancer.

However, Wallis’ meta-analysis of eight studies with 157,239 participants found an increased incidence of rectal cancer after radiotherapy for prostate cancer in unadjusted analysis. Moreover, a 5-year analysis of three studies with 204,064 patients found no statistically significant association, noting that there was considerable heterogeneity between studies and between radiotherapy techniques [45].

The latency period between radiotherapy performed for a primary site and a second malignancy occurring in the treated volume is estimated on average at 10 years after radiotherapy [45].

Also, the implications of a high proportion of urological second primary sites (bladder or renal) suggest possible shared etiologic factors that are worth emphasizing in follow-up strategies.

We observed a higher Gleason score in patients who developed metachronous prostate cancer. It is possible that previous therapies for the first neoplastic site used resistant clones.

These tumors are also characterized by genomic instability, and studies show that patients who later develop prostate cancer may have more aggressive forms.

In this regard, patients who have been treated for a primary neoplasm are recommended to be monitored to detect relapses, to monitor complications of oncological treatments, and to detect the appearance of a second cancer.

A study published in 2019, analyzing 30,964 patients, found that in patients with prostate cancer in Taiwan, the risk of developing a second neoplasm was lower for the lung, while patients younger than 64 years of age had a higher risk of thyroid cancer [46] (Table 10).

This article highlights the need for future studies regarding the management of synchronous/metachronous prostate cancer when other cancer sites are involved.

A further direction of investigation is related to patient no. 20, who presents a BRCA (breast cancer gene) mutation and consequently might have a hormonal component, and also related to patients no. 17 and 21, for whom the second cancer site may have been radio-induced or not.

We propose genetic susceptibility (BRCA1/2, HOXB13, and mismatch repair genes), therapy-related mutagenesis, hormonal interactions, and environmental exposures prevalent in Romania for future analyses.

The limitations of the study are the small sample with no comparison group (regarding the risk of developing bladder, colon, or rectal cancer in prostate cancer patients who have undergone prostatectomy versus definitive radiotherapy.); the fact that the study was conducted in a single medical facility and the patients were treated by means of various radiotherapy techniques; the lack of molecular profiling and the lack of BRCA testing.

## 5. Conclusions

This series represents a cohort study regarding synchronous/metachronous prostate cancer with other cancer sites. There are few reports on the management of these patients and there is a need to optimize clinical strategies for this complex patient group. This case series adds more experience to the already existing experience in the literature, providing guidelines in making decisions regarding the monitoring and treatment of the diseases. Our study revealed that treatment must combine the known multimodal therapy for each cancer. The therapeutic strategy of these tumors does not differ from the one of patients with a single tumor, but we must take into account that the sample size was small. As for future directions, considering that prostate cancer is a frequent location and with long survival, with different treatments depending on the stage of the disease, the genomic profile, the life expectancy, and the patient’s options, including different radiotherapy techniques introduced in the past decade (SBRT), and considering the potential risk of the appearance of the second neoplasm in the irradiated volume, we propose performing molecular biomarker analysis (BRCA 1, BRCA 2, CHEK 2, and CDK12) in metastatic prostate cancer patients and genetic testing in genetic/familial cancers. In patients who have a history of radiotherapy for prostate cancer, we suggest colonoscopy every 2–3 years.

What we propose is the careful monitoring (clinical examination, imaging investigations, PSA determination, and colonoscopy) and the assessment of individual risks based on genetic factors, treatments performed, and the lifestyle of patients with localized disease even 5 years after the completion of treatments.

## Figures and Tables

**Table 1:** Clinical and therapeutic parametrics.

Clinical and Therapeutic Parametrics	No. of Patients (%)
Median age (range) (years)	72.5 (56–83)
**First tumor site**	
- Prostate	14 (53.86)
- Bladder	2 (7.69)
- Kidney	3 (11.54)
- Colon-rectum	2 (7.69)
- Breast	1 (3.85)
- Larynx	2 (7.69)
- Liver	1 (3.85)
- Nasopharynx	1 (3.85)
**Second tumor site**	
- Prostate	10 (38.46)
- Lung	4 (15.38)
- Bladder	2 (7.69)
- Colon-rectum	5 (19.23)
- Stromal gastric tumor	1 (3.85)
- Thyroid	2 (7.69)
- Kidney	1 (3.85)
- Soft tissues	1 (3.85)
**Third tumor site**	
- Prostate	2 (7.69)
- Colon-rectum	2 (7.69)
- Pancreas	1 (3.85)

**Table 2:** The first primary cancer site.

Patient No.	Diagnosis	HP (Histopathological Exam); Molecular Markers	Year	Stage of the Disease	Treatment		
1	Prostate	Adenocarcinoma Gleason 8 (4 + 4)	2022	IV		EBRT	ADT + ARPI (Abiraterone)
2	Prostate	Adenocarcinoma Gleason 7 (3 + 4)	2022	III	Surgery		
3	Prostate	Adenocarcinoma G2 Gleason 6 (3 + 3)	2019	III		EBRT	ADT + ARPI (Apalutamide)
4	Prostate	Adenocarcinoma Gleason 7 (3 + 4)	2023	II		EBRT	ADT
5	Prostate	Adenocarcinoma Gleason 7 (3 + 4)	2023	III		EBRT	
6	Prostate	Adenocarcinoma Gleason 7 (4 + 3)	2021	IV		EBRT	ADT
7	Prostate	Adenocarcinoma Gleason 7 (4 + 3)	2024	II	Surgery		
8	Larynx	Squamous carcinoma G1	2011	I	Surgery		
9	Colon	Adenocarcinoma	2014	II	Surgery		
10	Prostate	Adenocarcinoma Gleason 6 (3 + 3)	2016	III			ADT
11	Prostate	Adenocarcinoma Gleason 6 (3 + 3)	2018	II		EBRT	ADT
12	Kidney	Clear cell carcinoma	2015	IV	Surgery		
13	Larynx	Squamous cell carcinoma	2013	III	Surgery	EBRT adjuvant	
14	Bladder	Papillary carcinoma	2010	II	TUR-V		Instillation CHT
15	Colon	adenocarcinoma	2022	II	Surgery		CHT
16	Prostate	Adenocarcinoma Gleason 9 (4 + 5)	2021	IV		EBRT	ADT
17	Prostate	Adenocarcinoma Gleason 8 (3 + 5)	2014	IV		EBRT	ADT (36 months)
18	Kidney	Oncocytoma	2010	I	Surgery		
19	Prostate	Adenocarcinoma Gleason 5 (2 + 3)	2010	II		EBRT	ADT
20	Breast	Invasive breast carcinoma Luminal B, RE-80%, PR-30%, HER2-negative KI 67 25%	2015	II	Surgery	EBRT	CHT, HT (adjuvant, 5 years)
21	Bladder	Urothelial carcinoma	2015	II	TUR-V	EBRT at relapse	
22	Prostate	Adenocarcinoma Gleason 8 (4 + 4)	2023	III		EBRT at relapse	ADT
23	Liver	Unresectable carcinoma	2018				Chemoembolization, Sorafenib
24	Prostate	Adenocarcinoma Gleason 7 (4 + 3)	2018	III		EBRT	
25	Nasopharynx	Carcinoma	2010	II		EBRT	
26	Kidney	Clear cell carcinoma	2020	III	Surgery		

Abbreviations: ER = estrogen receptor; PR = progesterone receptor; HER2 = human epidermal growth factor; EBRT = external beam radiotherapy; HT = hormonal therapy; ARPI = androgen receptor pathway inhibitors); ADT = androgen deprivation therapy; CHT = chemotherapy; TURBT = Transurethral resection of bladder tumor. Patient no./Diagnosis/Histopathological exam/Year of diagnosis/The stage of the disease (nonmetastatic/metastatic)/Treatment (Surgery/EBRT /CHT/ADT/TKI = cellular marker for proliferation).

**Table 3:** The second primary cancer site.

Patient No.	Diagnosis	HP (Histopathological Exam); Molecular Markers	Year	Stage	Treatment		
1	Lung	Adenocarcinoma EGFR-negative, ALK-negative, PDL1-negative	2023	III	Surgery		CHT
2	Lung	Large cell, EGFR-negative, ALK-negative, PDL1-negative	2024	IV			CHT
3	Bladder	Papillary carcinoma	2024	I	Surgery		
4	Lung	Adenocarcinoma EGFR-negative, ALK-negative, PDLI-negative	2024	III	Surgery		CHT
5	Lung	Adenocarcinoma G2, EGFR-positive, ALK-negative	2024	III			Anti-EGFR therapy-Osimertinib
6	Colon	Adenocarcinoma	2024	III	Surgery		
7.	Bladder	Urothelial carcinoma	2024	II	TUR-V		CHT
8.	Prostate	Adenocarcinoma Gleason 6 (3 + 3)	2021	II-INTERMEDIATE GROUP RISK		EBRT	ADT
9	Prostate	Adenocarcinoma Gleason 9 (4 + 5)	2022	II		EBRT	ADT + ARPI (Enzalutamide)
10	Colon	Adenocarcinoma	2024	II	Surgery		
11	GIST Stomach	GIST	2018		Surgery		Imatinib
12	Prostate	Adenocarcinoma Gleason 8 (4 + 4)	2022	II		EBRT	ADT
13	Prostate	Adenocarcinoma Gleason 6 (3 + 3)	2016	III		EBRT	ADT (36 months)
14	Prostate	Adenocarcinoma Gleason 7 (3 + 4)	2017	II	Surgery		
15	Prostate	Adenocarcinoma Gleason 9 (4 + 5)	2024	IV		EBRT	ADT + ARPI (Apalutamide)
16	Colon	Adenocarcinoma	2024	I	Surgery		
17	Rectum	Adenocarcinoma	2024	III	Surgery		CHT
18	Thyroid	Papillary carcinoma	2019		Surgery-thyroidectomy	Radioactive Iodine Therapy	Sorafenib
19	Sarcoma	Leiomyosarcoma	2014		Surgery	adjuvant EBRT	
20	Prostate	Adenocarcinoma Gleason 6 (3 + 3)	2023	IV		EBRT	ADT
21	Prostate	Adenocarcinoma Gleason 6 (3 + 3)	2024	IV		EBRT	ADT
22	Rectum	Adenocarcinoma	2024	III	Surgery	EBRT	CHT
23	Prostate	Adenocarcinoma Gleason 7 (4 + 3)	2023	II	Surgery		ADT (36 months)
24	Thyroid	Papillary carcinoma	2019		Surgery		
25	Kidney	Clear cell carcinoma	2016	II	Surgery		
26	Prostate	Adenocarcinoma Gleason 9 (4 + 5)	2023	IV		EBRT	ADT

Abbreviations: GIST = Gastrointestinal Stromal Tumor. Patient no./Diagnosis/Histopathological exam/Year of diagnosis/Stage of the disease (nonmetastatic/metastatic)/Treatment (Surgery/EBRT/CHT/ADT/TKI = tyrosine kinase inhibitor).

**Table 4:** The third primary cancer site (five patients).

Patient No.	Diagnosis	HP (Histopathological Exam)	Year	Stage	Treatment		
5	Rectum	Adenocarcinoma	2025	IV		EBRT	
18	Prostate	Adenocarcinoma Gleason 9 (4 + 5)	2025	IV		EBRT	ADT
19	Colon	Adenocarcinoma	2021	II	Surgery		
24	Pancreas	Adenocarcinoma	2021	II	Surgery		
25	Prostate	Adenocarcinoma Gleason 7 (4 + 3)	2020	IV		EBRT	ADT

**Table 5:** The most frequent combinations.

No.	Combination	No. of Cases
1	Prostate Cancer plus Bladder/Renal Cancer	7
2	Prostate Cancer plus Colon/Rectum	7
3	Prostate Cancer plus Two Cancers	5
4	Prostate Cancer plus Lung Cancer	4

**Table 6:** Time period (months) between the first and the second cancer site occurrence. Synchronous/metachronous cancer.

Patient No.	Time Period (Months) Between the First and the Second Cancer Site Occurrence	Synchronous/Metachronous Cancer
1	12	metachronous
2	24	metachronous
3	60	metachronous
4	12	metachronous
5	3	synchronous
6	36	metachronous
7	4	synchronous
8	120	metachronous
9	96	metachronous
10	96	metachronous
11	8	metachronous
12	84	metachronous
13	36	metachronous
14	84	metachronous
15	24	metachronous
16	36	metachronous
17	120	metachronous
18	108	metachronous
19	48	metachronous
20	96	metachronous
21	108	metachronous
22	12	metachronous
23	60	metachronous
24	12	metachronous
25	72	metachronous
26	36	metachronous

**Table 7:** Time period (months) between the first and the third cancer site occurrence. Synchronous/metachronous (five patients).

Patient No.	Time Period (Months) Between the First and the Third Cancer Site Occurrence	Synchronous/Metachronous
5	24	metachronous
18	180	metachronous
19	132	metachronous
24	36	metachronous
25	120	metachronous

**Table 8:** Time period (months) between the second and the third cancer site occurrence. Synchronous/metachronous (five patients).

Patient No.	Time Period (Months) Between the Second and the Third Cancer Site Occurrence	Synchronous/Metachronous
5	12	metachronous
18	72	metachronous
19	84	metachronous
24	24	metachronous
25	48	metachronous

**Table 9:** Regression summary for survival.

Regression Summary for Survival					
Predictor	Standard Error	t Stat	*p*-Value	95% Lower	95% Upper
Intercept	0.518	1.717	0.1001	−0.185	1.964
Gleason	0.0713	0.076	0.9397	−0.142	0.153
Prostate tumor_P1	0.0954	0.687	0.8832	−0.098	0.154
Prostate tumor_P2	0.151	0.477	0.6381	−0.242	0.386
Prostate tumor_P3	0.277	0.251	0.8042	−0.505	0.644

**Table 10:** Comparative analytical data.

No.	Author, Year	Subject: Prostate Cancer/Other Cancer; Synchronous/Metachronous	Reference No.
1	Zhang Y et al., 2024	Prostate/renal; case report; synchronous	[27]
2	Bunnag N et al., 2023	Prostate/lung; case report; synchronous	[28]
3	Sarkar S et al., 2021	Prostate/Lung; case report; synchronous	[29]
4	Luqman W et al., 2020	Prostate/sarcoma; case report; synchronous	[30]
5	Edfelt E et al., 2025	Prostate/rectum; large cohort study; synchronous	[31]
6	B.U. Sidiqi et al., 2023	Prostate/rectum; case series (10 cases); synchronous	[32]
7	Sossa J et al., 2023	Prostate/breast; case report; metachronous	[33]
8	Millican J et al., 2022	Prostate/rectum; single-institution experience; synchronous/metachronous	[40]
9	Hoshi S et al., 2024	Prostate/urothelial; three cases; metachronous	[40]
10	Zhao T et al., 2021	Kidney/prostate/lung; case report; metachronous	[41]
11	Omer DM et al., 2023	Rectal cancer after prostate radiation; synchronous/metachronous	[44]
12	Neugut AI et al., 1997	Second malignancies after radiotherapy for prostate carcinoma; synchronous/metachronous	[45]
13	Wallis CJ et al., 2019	Second malignancies after radiotherapy for prostate cancer; synchronous/metachronous	[46]

## Data Availability

The data presented in this study are available on request from the corresponding author due to privacy policy. These data are collected from the medical files of patients and might be available if requested.

## Abbreviations

The following abbreviations are used in this manuscript:

BRCA2	breast cancer-related gene 2
MPMs	multiple primary malignancies
SEER	Surveillance, Epidemiology, and End Results Program
IACR/IARC	International Association of Cancer Registries and International Agency for Research on Cancer
EBRT	external beam radiotherapy
ADT	androgen deprivation therapy
CHT	chemotherapy
TKI	tyrosine kinase inhibitor
GIST	Gastrointestinal Stromal Tumor
ARPI	Androgen receptor pathway inhibitors
